# Impact of multiplex PCR point-of-care platform implementation for respiratory pathogen detection in an emergency department with high daily patient volume

**DOI:** 10.1128/jcm.01313-25

**Published:** 2025-12-01

**Authors:** Benjamin Bigaud, Nicolas Marjanovic, Luc Deroche, Bertrand Drugeon, Marvin Piot, Nicolas Leveque, Olivier Mimoz, Jérémy Guenezan

**Affiliations:** 1 Service des Urgences SAMU SMUR, CHU Poitiers 36655, Poitiers, France; 2 INSERM U1070, Pharmacologie des Agents Anti-Infectieux et Résistance (PHAR2), Poitiers, France; 3 Laboratoire de virologie et mycobactériologie, CHU de Poitiers 36655 , Poitiers, France; 4 Laboratoire Inflammation Tissus Épithéliaux et Cytokines, Université de Poitiers 27077, Poitiers, France; 5 INSERM U1313, Ischémie Reperfusion, Métabolisme et Inflammation Stérile en Transplantation (IRMETIST), Poitiers, France.; 6 Alliance for Vascular Access Teaching and Research (AVATAR) group, Griffith University 5723, Brisbane, Queensland, Australia; 7 Point of Care in Emergency Room (POCER) Network, ANR-24-DIME-0003, CHU de Bordeaux, Bordeaux, France; Maine Medical Center Department of Medicine, Portland, Maine, USA

**Keywords:** respiratory tract infections, syndromic testing, antimicrobial stewardship, diagnostic stewardship, turnaround time, isolation precautions

## Abstract

Lower respiratory tract infections (LRTIs) place a substantial burden on emergency departments (EDs) during winter outbreaks. Their microbiological diagnosis is currently based on either broad-spectrum molecular techniques performed in laboratory, with turnaround times incompatible with ED workflows, or point-of-care systems, restricted to SARS-CoV-2, influenza A/B, and RSV (quadriplex panel). The Spotfire combines rapid result and syndromic approach (10 respiratory viruses and 4 atypical bacteria), but its added value in EDs remains unassessed. During the 2023 winter, trained nurses collected nasopharyngeal swabs from adult patients with LRTI symptoms and operated the Spotfire in the ED of the Poitiers University Hospital. We described the viral epidemiology and evaluated the impact and efficacy of this rapid broad-spectrum diagnosis by comparing the management of patients with or without respiratory pathogen detected. From 15 December 2023 to 15 March 2024, 1,320 samples were analyzed with Spotfire, with a median turnaround time of 37 min and 10 uninterpretable results. A total of 540 (41%) were positive. Influenza A (30.9%), rhinovirus/enterovirus (21.1%), and SARS-CoV-2 (18.7%) were the main viruses detected, while atypical bacteria represented 8% of all pathogens. Overall, a pathogen not represented on the quadriplex panel was detected in 43% samples. Positive test results were associated with faster medical decision (380 ± 234 vs 431 ± 238 min; *P* < 0.001), fewer hospital admissions (65% vs 78%; *P* < 0.001), and shorter hospital stay (10 ± 9 vs 12 ± 14 days; *P* = 0.006) than negative test results. Antibiotic therapy was administered to 42 of 43 of *Mycoplasma pneumoniae*-positive patients (98%) compared with 110 of 1,265 (9%) negative patients. This study validated the feasibility of this new diagnostic tool in high-volume EDs improving patient flow, antimicrobial decisions, and isolation strategies.

**IMPORTANCE:**

This study provides the first real-world evidence supporting the use of a broad multiplex PCR platform for respiratory pathogens directly at the point of care in a high-volume emergency department. By enabling the simultaneous detection of 14 viruses and atypical bacteria within 20 min, this system bridges a critical gap between laboratory diagnostics and bedside clinical decision-making. Its implementation proved feasible and reliable, improving patient flow, antimicrobial stewardship, and infection control measures. Nearly half of the pathogens identified would have been missed by conventional quadriplex assays, highlighting the added diagnostic value of broader syndromic coverage. These findings are of interest to both clinicians and microbiologists, as they provide pragmatic evidence to guide the integration of advanced molecular diagnostics into acute-care workflows and to optimize patient management during respiratory infection surges.

## INTRODUCTION

Lower respiratory tract infections (LRTIs) are among the leading causes of morbidity and mortality worldwide (1–3). During outbreak periods, they place a significant strain on the healthcare system and, especially, on emergency departments (EDs), due to a marked increase in LRTI-related visits (4). As EDs must perform rapid triage and initiate appropriate treatment under time pressure, managing patients with LRTIs remains challenging, particularly in terms of optimizing care and improving patient outcomes.

LRTIs have diverse microbial etiologies. Typical bacteria, such as *Streptococcus pneumoniae* and *Haemophilus influenzae*, account for approximately 11% of documented pathogens, while atypical bacteria (*Mycoplasma pneumoniae*, *Chlamydia pneumoniae*, *Bordetella pertussis*) all together are involved in 13% (5). Viral pathogens, which constitute the majority of LRTIs etiologies (53%), include influenza (flu) A/B, respiratory syncytial virus (RSV), rhinoviruses/enteroviruses, severe acute respiratory syndrome-related coronavirus 2 (SARS-CoV-2), seasonal coronaviruses, parainfluenza viruses, and human metapneumovirus, with prevalence varying depending on the month, year, and population (adult or pediatric) (2).

Lower respiratory tract specimens (e.g., sputum or tracheal aspirates) are generally recommended for etiological confirmation in patients with suspected LRTIs, particularly in severe cases (6). However, nasopharyngeal sampling remains the most practical and standardized approach in the emergency department for the entire population. Several studies have shown good agreement between upper and lower respiratory specimens for molecular pathogen detection in this setting (7). Therefore, multiplex molecular tests are now routinely used for the microbiological diagnosis of LRTIs. Performed from nasopharyngeal swabs, they detect, with excellent sensitivity and specificity, a wide range of viruses and atypical bacteria (8). However, despite the development of laboratory-based multiplex molecular assays providing results within a few hours, they are still, in many institutions, processed in main laboratories, in series, once a day, during opening hours, resulting in turnaround times that remain incompatible with ED workflows. Several randomized and controlled trials have demonstrated the clinical value of molecular point-of-care (POC) testing in acute-care settings, including faster turnaround times and shorter ED stays (9), improved infection control and isolation practices (10, 11), and more appropriate antimicrobial use (12, 13). Rapid PCR testing in adults hospitalized with respiratory illness has also been associated with patient benefits and reduced resource utilization (14). However, most currently available POC platforms are limited in scope, often restricted to singleplex or quadriplex panels targeting only SARS-CoV-2, influenza A/B, and RSV. While these tests typically deliver results within a few minutes, their narrow panel may necessitate additional investigations when initial results are negative or alternative etiologies are suspected. To overcome this limitation, integrated molecular platforms have been recently designed to combine the rapidity and decentralization of POC testing with the diagnostic breadth of multiplex panels identical to that practiced in virology laboratories. The Spotfire platform (bioMérieux, Marcy-l'Étoile, France) is a cartridge-based, fully automated multiplex PCR platform capable of detecting 10 respiratory viruses and four atypical bacteria in approximately 20 min. Its high analytical performance, confirmed by both manufacturer data and independent evaluations (15), supports its potential integration into ED workflows.

We hypothesized that deploying this molecular platform in routine ED workflows would be both feasible and beneficial for clinical pathways. Accordingly, the primary objective of this study was to evaluate, for the first time, the feasibility of the Spotfire platform as a POC in a high-volume daily patient ED during the 2023 winter LRTI outbreak period. We also aimed to assess its impact on patient management, isolation, antibiotic and additional test prescriptions, and, overall, estimate its potential benefit compared with quadriplex POC use.

## MATERIALS AND METHODS

### Setting

We conducted a single-center, retrospective study at the Poitiers University Hospital in France, with 1,000 hospital beds whose 50% double bedrooms (limiting the possibilities for isolating symptomatic patients) and one adult ED handling approximately 50,000 visits a year. Since 2023, the hospital made the Spotfire available to the ED during each winter LRTI outbreak period.

### Patients

Patients with the following criteria were eligible in the study: age equal to or over 15 years and 3 months (threshold for adult ED admission in our institution); visiting the adult ED between 7 December 2023 and 15 March 2024, corresponding to the 2023–2024 winter LRTI outbreak period; presenting lower respiratory tract symptoms, such as cough, expectoration, shortness of breath, chest pain upon breathing or coughing, and general signs, such as fever or fatigue (16, 17); requiring hospitalization for any reason (not limited to respiratory infection) or for whom the nasopharyngeal test result would have a significant impact on clinical management. In line with national guidelines (18), these conditions included patients with immunocompromised status; patients with suspected atypical bacterial pneumonia (*Mycoplasma pneumoniae*, *Chlamydia pneumoniae*, *Bordetella pertussis*); or situations where pathogen identification could influence decisions regarding prescription of additional tests, antibiotic treatment initiation or discontinuation, or implementation of isolation precautions (e.g., allocation to single or double room). Of note, this study period also coincided with an unusual post-pandemic resurgence of *Mycoplasma pneumoniae* infections across France (19). In patients who had more than one Spotfire test during the same ED visit or during different ED visits, only the first test was kept for analysis.

### Clinical staff training

To ensure proper implementation of the Spotfire platform within the ED workflow, a dedicated team of nurses received targeted training to operate the platform and perform associated tests. This training was conducted jointly by the microbiology laboratory staff and the bioMérieux technical team. The dedicated team received a 1-h theoretical and practical session covering all aspects of nasopharyngeal sample collection and operation of the Spotfire platform. The session included a review of manufacturer-provided instructions, live demonstrations, and supervised hands-on practice. Training adhered to the standard operating procedures in place at our institution and ensured that nurses could independently perform the test in compliance with both institutional and manufacturer guidelines.

### Nasopharyngeal swab handling

At the first medical contact, either the triage nurse or the emergency physician was responsible for identifying eligible patients. Once identified, the dedicated nursing team was contacted to perform the Spotfire test as early as possible. In some cases, the treating ED physician could request the test later during the patient’s evaluation.

Nurses dedicated to the Spotfire test first collected the nasopharyngeal sample using a standard flocked swab thoroughly homogenized into inactivating transport medium containing a chemical lysis buffer to inactivate pathogens and stabilize nucleic acids before analysis (SunTrine Biotechnology, Jiangsu, China), following institutional protocols. They then performed the Spotfire R panel (BIOFIRE Diagnostics, Salt Lake City, UT, USA) on the Spotfire System according to the manufacturer’s recommendations. This POC platform allowed for the nucleic acid detection of 10 viruses (adenovirus, SARS-CoV-2, seasonal coronavirus, human metapneumovirus, human rhinovirus/enterovirus, influenza A virus with A/H1-2009, and A/H3 subtyping, influenza B virus, parainfluenza virus, and RSV) and four bacteria (*Bordetella parapertussis, Bordetella pertussis, Chlamydia pneumoniae,* and *Mycoplasma pneumoniae*) within 20 min. In the event of an initial failed result, the protocol specified that a single repeat analysis of the same sample should be performed before considering sending the specimen to the main laboratory.

The trained nurses were available daily in the ED, from 10 a.m. to 10 p.m., 7 days a week, ensuring that testing could be conducted efficiently during peak patient periods. Outside of the dedicated team working hours, nasopharyngeal sampling was performed by ED nurses using the same technique and sent to the main laboratory, where the Allplex Respiratory Panel assay (Seegene) was used for pathogen detection. In such cases, results were not available in real time but were typically returned within 48 hours.

### Additional tests and antibiotic therapy

As this was a retrospective study, the choice of performing additional tests, such as blood sampling for biological analyses, other bacteriological tests (blood cultures, urinary antigen tests...) to document LRTI or other infection sites, or imaging tests (chest X-ray or computed tomography [CT] scan) was left to the discretion of the ED physician, following national guidelines (20).

### Outcomes

The primary outcome was the feasibility of implementing the Spotfire platform in real-life ED setting. To evaluate this, we extracted the following from the microbiology laboratory database during the Spotfire platform availability period: the number of tests performed; number of failed results; number of nasopharyngeal samples directly sent to the main laboratory for respiratory pathogen testing during the Spotfire platform availability period; and overall turnaround time (TAT), defined as the delay between test prescription and result availability to the clinician. In addition, we conducted a descriptive analysis of the tested population and the pathogens detected to further characterize real-world implementation.

Secondary outcomes were to compare the following between patients with positive or negative Spotfire tests: (i) need for respiratory support in the ED; (ii) time from ED admission to medical decision; (iii) need for hospital admission; (iv) hospital length of stay; (v) 30-day mortality; and (vi) prescription of additional laboratory and imaging investigations to explore the impact of rapid pathogen detection on patient trajectories and decision-making pathways. We also looked at the impact of using the Spotfire platform on the administration of appropriate antibiotic therapy against atypical bacterial pathogens (i.e., macrolide or fluoroquinolone).

Additionally, we assessed the potential added value of using the broad spectrum of the Spotfire platform instead of a usual quadriplex panel restricted to SARS-CoV-2, influenza A/B, and RSV only, often considered as the first-line panel in national and international recommendations, by determining (i) the number of patients and pathogens detected by the Spotfire that would have been missed by quadriplex panels and (ii) the potential impact on isolation precautions (hospital admission of patients carrying viruses in single room or cohorting in double room).

Also, in order to justify the use of a broad virological panel, we compared the severity of clinical condition (assessed by the risk of hospital admission and 30-day mortality) of positive patients to commonly targeted viruses with quadriplex panels versus positive patients to other respiratory viruses or atypical bacteria detected with the Spotfire platform. Patients with positive Spotfire tests were assigned into one of three groups. (i) The quadriplex viruses (QV) group included all patients in whom at least one virus from commonly used quadriplex panels was detected, regardless of co-infection with other pathogens. (ii) The atypical bacteria (AB) group included patients with detection of at least one atypical bacterial pathogen (*Mycoplasma pneumoniae*, *Chlamydia pneumoniae*, or *Bordetella pertussis*), with or without co-infection with non-quadriplex viruses. (iii) The non-quadriplex viruses (NQV) group included patients with respiratory viruses not covered by quadriplex panels and with no co-detection of QV viruses or atypical bacteria.

### Data collection

Cases were identified using the microbiology laboratory database of our institution listing the results of all Spotfire tests performed. Clinical, biological, and radiological data were retrospectively extracted from electronic medical records by an ED physician. Comorbidities were retrieved based on the documented medical history at the time of ED presentation. The following variables were collected:

- Demographics: age, gender.
- Comorbidities: hypertension, chronic heart failure, chronic respiratory failure, chronic kidney disease, diabetes, and immunosuppression.
- Laboratory and radiological tests: complete blood count, serum electrolytes, creatinine level, D-dimer, lactate, arterial blood gases, pneumococcal or legionella urinary antigen test, blood culture, chest X-ray, and chest CT scan.
- Respiratory support during the ED stay: oxygen therapy, high-flow oxygen therapy, and non-invasive or invasive ventilation.
- Antibiotic therapy prescribed in the ED: antibiotic prescriptions status and type of antibiotic prescribed.
- Outcome: hospital admission and type of room (single, cohorting in double room), length of stay in ED and hospital, and all-cause D-30 mortality.

### Statistical analyses

Quantitative data are reported as median and interquartile range, and qualitative data as number and proportion. Data were compared between study groups using Mann Whitney U-test or χ^2^ test, depending on variable type and distribution. Effect sizes are reported either as odds ratios (ORs) with their 95% confidence intervals (95% CIs), or as absolute differences with 95% CIs, depending on the nature of the outcome. We used the R statistical package version 3.6.2 or later (The R Foundation for Statistical Computing, https://www.R-project.org/). A *P*-value < 0.05 was considered significant. No imputation was performed for missing data, and the analyses were conducted on available cases.

Antibiotic prescription patterns were also analyzed by calculating the overall prescription rate and stratifying it according to the identified pathogen, in order to explore associations between microbiological results and antimicrobial use.

## RESULTS

### Study population

**Fig 1:**
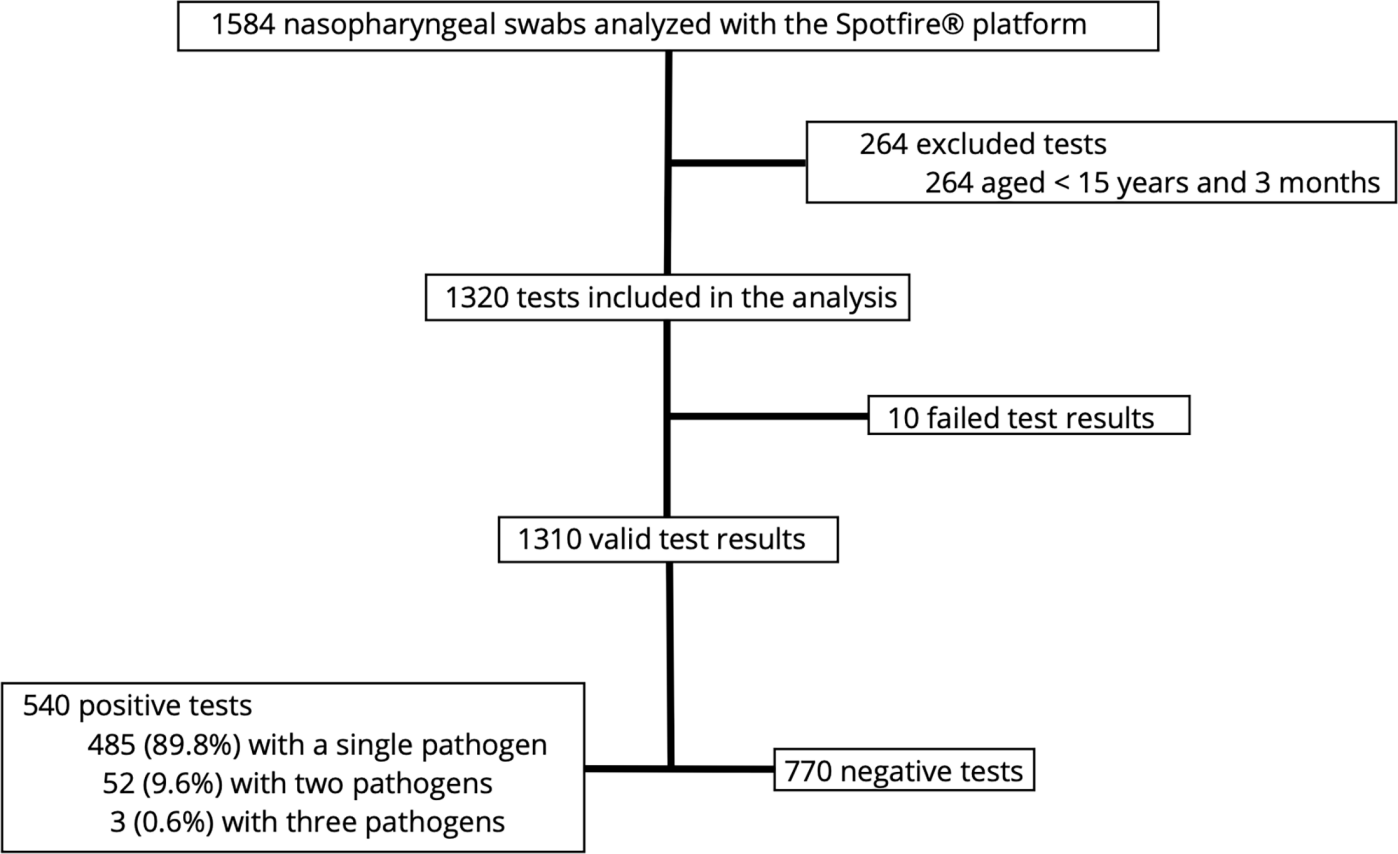
Study flowchart.

From 7 December 2023 to 15 March 2024 (corresponding to the 2023 winter LRTI outbreak period), 1,584 nasopharyngeal swabs from 1,584 patients visiting the ED with clinical picture of LRTI were tested using the Spotfire platform, representing approximately 10.3% of all 15,436 ED visits during the study period. Among these, 264 patients did not meet age inclusion criteria and were excluded, and 10 tests performed in eligible patients provided failed results despite two attempts. None of the included patients underwent second Spotfire testing during a distinct ED visit during the study period. Finally, 1,310 nasopharyngeal swabs from 1,310 patients were included and analyzed (Fig. 1). Among these patients, 685 (52%) were male, and their median age was 73 (56–85) years (Table 1). Hypertension (700 [53%] patients), chronic respiratory disease (291 [22%]), and diabetes (253 [19%]) were the most common comorbidities.

**TABLE 1:** Baseline characteristics of the study population according to Spotfire test results^*a*^

Characteristics	All patients (*n* = 1,310)	Positive (*n* = 540)	Negative (*n* = 770)	*P*-value
Male sex	685 (52)	270 (50)	415 (54)	0.2
Female sex	625 (48)	270 (50)	355 (46)	0.2
Age, yr (median [IQR])	73 (56–85)	69 (50–84)	75 (61–86)	**<0.001**
Comorbidities^*b*^				
Hypertension	700 (53)	268 (50)	432 (56)	**0.021**
Chronic respiratory disease	291 (22)	115 (21)	176 (23)	0.5
Diabetes (type 1 or 2)	253 (19)	111 (21)	142 (18)	0.3
Heart failure	131 (10)	39 (7.2)	92 (12)	**0.005**
Chronic renal failure	104 (7.9)	38 (7.0)	66 (8.6)	0.3
Immunosuppression	49 (3.7)	25 (4.6)	24 (3.1)	0.2

^*a*^ Results are *n* (%) except where indicated: median (IQR). Bold *P*-values indicate statistically significant differences (*P* < 0.05).

^*b*^ One patient may have more than one comorbidity.

### Feasibility

Of the 1,320 eligible tests, 10 (0.8%) provided failed results despite repeated analysis, and the microbial sample should be sent to the main laboratory. Of the 1,310 tests that provided a valid result, 15 (1.1%) required a repeated analysis due to failed result at the first assessment. No patient required repeated sample collection, and none of the nasopharyngeal swabs collected during the availability period of the Spotfire platform was sent directly to the main laboratory for analysis following equipment failure. For patients with available data (*n* = 810; 61.8%), the median TAT between test prescription and result availability to the clinician was 37 min (IQR 29.3–53.0).

### Pathogens detected and epidemiology

**Fig 2:**
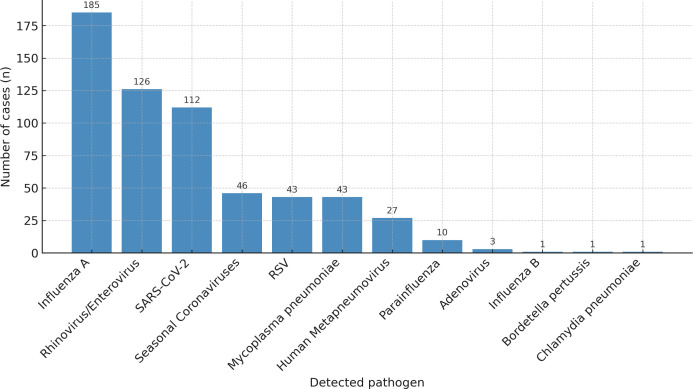
Distribution of respiratory pathogens detected by the Spotfire multiplex PCR platform. Distribution of the 598 respiratory pathogens identified among the 540 patients who were tested positive using the Spotfire platform. Influenza A, rhinovirus/enterovirus, and SARS-CoV-2 were the most frequently detected viruses. Atypical bacterial pathogens—including *Mycoplasma pneumoniae*, *Bordetella pertussis*, and *Chlamydia pneumoniae*—accounted for 45 detections (7.5%). Co-detections of two or more pathogens occurred in 10.2% of cases (not shown in the figure). RSV stands for respiratory syncytial virus and SARS-CoV-2 for severe acute respiratory syndrome coronavirus 2.

The Spotfire test was positive for 540 of 1,310 (41.2%) swabs, enabling the identification of 598 pathogens. A single pathogen was identified in 485 (89.8%) swabs, 2 in 52 (9.6%) and 3 in 3 (0.6%) (Tables S1 to S4). The most frequently detected virus was influenza A (185 cases, 30.9% of all pathogens; 34.3% of patients), followed by rhinovirus/enterovirus (126 cases, 21.1%; 23.3%) and SARS-CoV-2 (112 cases, 18.7%, 20.7%) (Fig. 2 and Table S1l). Other respiratory viruses represented less than 10% of all pathogens. Bacteria represented 45 (7.5%) of all pathogens and were identified in 45 (8.3%) patients. *Mycoplasma pneumoniae* accounted for 43 cases, while *Bordetella pertussis and Chlamydia pneumoniae* were detected in one sample each.

**Fig 3:**
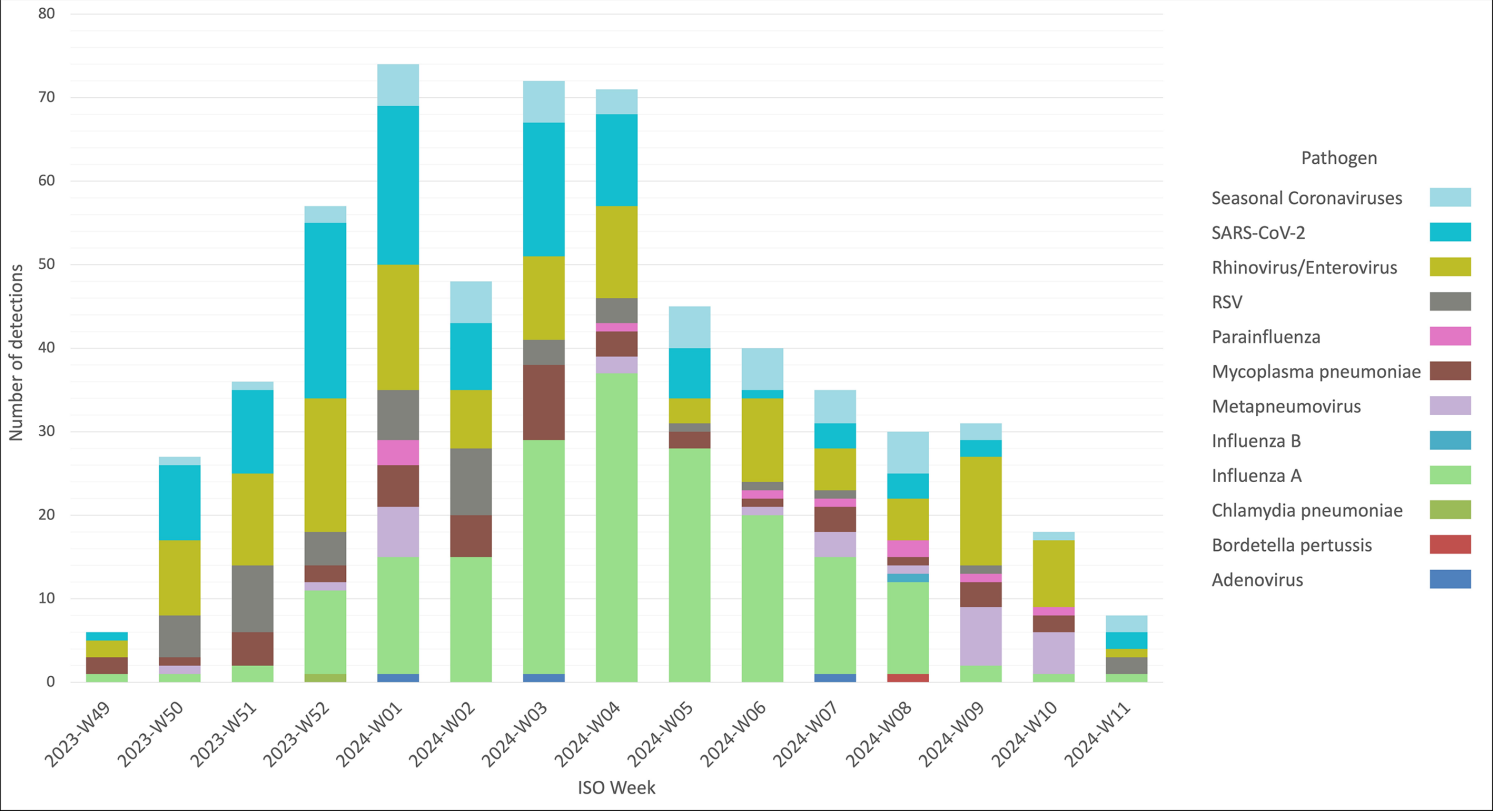
Weekly incidence of respiratory pathogens detected by the Spotfire multiplex PCR platform during the study period (ISO weeks). Temporal distribution of the 598 respiratory pathogens detected among 540 positive patients, presented by ISO week (W49-2023 to W11-2024). The most prominent viral peaks included SARS-CoV-2 (weeks W52–W01) and influenza A virus (week W04), while other pathogens, such as rhinoviruses/enteroviruses, seasonal coronaviruses, and atypical bacteria (e.g., *Mycoplasma pneumoniae*) circulated more diffusely. Each color represents one pathogen, as indicated in the legend. Co-infections are represented as separate detections. RSV stands for respiratory syncytial virus and SARS-CoV-2 for severe acute respiratory syndrome coronavirus 2.

The distribution of respiratory pathogens varied across the study period (Fig. 3). Influenza A activity peaked in January, following a moderate increase in December and declining in February. Human rhinovirus/enterovirus circulation was observed throughout the period, with small peaks in early and late winter. SARS-CoV-2 showed a marked peak in December and January. Other viruses, such as RSV and human metapneumovirus, exhibited a more heterogeneous temporal distribution, with lower and more scattered circulation across the winter months. Co-infections (≥2 pathogens) were observed throughout the period, with higher frequencies coinciding with the peak viral activity.

### Secondary outcomes

Compared with patients with negative Spotfire test, patients with positive test required oxygen therapy more frequently (56% vs 48%, absolute difference 8 [95%CI 2.8 to 13.8]%; *P* = 0.004), had shorter time from admission to medical decision in the ED (380 ± 234 vs 431 ± 238 min; mean difference 51 [−77 to −25] min; *P* < 0.001), required hospital admission less frequently (65% vs 78%; absolute difference −13 [−18 to −8.2]%; *P* < 0.001), had shorter hospital length of stay (10 ± 9 vs 12.1 ± 14 days; mean difference −2.1 [−3.5 to −0.6] days; *P* = 0.006) and were at lower risk of death (7.8% vs 14%, absolute difference −6.2 [−10 to −2.4]%; *P* < 0.001) (Table 2). The need for invasive or noninvasive ventilation in the ED was similar between groups. Regarding additional investigations, patients with positive Spotfire tests were less frequently prescribed complete blood count (86% vs 94%; absolute difference: −7.2% [−10.5 to −3.8]; *P* < 0.001) and serum electrolytes/creatinine testing (86% vs 94%; −7.3% [−10.7 to −4.0]; *P* < 0.001), but more frequently prescribed D-dimer testing (19% vs 12%; +6.9% [+2.9 to +10.9]; *P* < 0.001) and chest X-ray (75% vs 69%; +5.9% [+0.9 to +10.8]; *P* = 0.021) than patients with negative Spotfire tests (Table S5). No difference was observed for lactate measurement, arterial blood gas, urinary antigen tests, and chest CT scan.

**TABLE 2:** Secondary outcomes according to Spotfire test result^*a*^

Characteristics	All patients (*n* = 1,310)	Positive tests (*n* = 540)	Negative tests (*n* = 770)	Absolute risk difference / mean difference [95% CI]	*P*-value
Respiratory support in the ED					
Oxygen therapy (via nasal cannula or face mask)	671 (51)	302 (56)	369 (48)	+8 [2.8 to 13.8]	**0.004**
High-flow oxygen therapy	4 (0.3)	3 (0.6)	1 (0.1)	—	0.3
Non-invasive ventilation	52 (4)	25 (4.6)	27 (3.5)	+1.1 [−0.8 to 3.0]	0.3
Mechanical ventilation	5 (0.4)	2 (0.4)	3 (0.4)	—	>0.9
Medical decision-making time, min	406 ± 239	380 ± 234	431 ± 238	−51 [−77 to −25]	**<0.001**
Follow-up					
Hospital admission	952 (73)	350 (65)	602 (78)	13 [−18 to −8.2]	**<0.001**
Short hospitalization unit	188 (20)	55 (16)	133 (22)	−6 [−10.9 to −1.1]	**0.017**
Medical ward	796 (83)	297 (84)	499 (82)	+2 [−2.2 to 5.9]	0.4
Intermediate or intensive care unit admission	68 (7.1)	26 (7.4)	42 (6.9)	+0.5 [−1.7 to 2.7]	0.8
Length of hospital stay, days	11.3 ± 12.4	10 ± 9	12.1 ± 14.0	−2.1 [−3.5 to −0.6]	**0.006**
30-day mortality	153 (12)	42 (7.8)	111 (14)	−6.2 [−10 to −2.4]	**<0.001**

^*a*^ Values are presented as *n* (%) or mean ± SD. Differences between groups are reported as absolute risk differences for categorical outcomes and as mean differences for continuous outcomes, both with 95% confidence intervals. ED denotes emergency department. Bold *P*-values indicate statistically significant differences (*P* < 0.05). — indicates that the absolute risk difference could not be calculated.

Among the 43 patients with atypical bacterial pathogen detected, 42 (97.7%) received targeted antibiotic therapy, and the only untreated case corresponded to an end-of-life patient for whom only comfort care was initiated. In contrast, among patients without detected atypical bacterial pathogens (*n* = 1,265), 110 (8.7%) received antibiotics active against these pathogens: 20 for non-respiratory infections requiring such treatment, and 90 without microbiological documentation, most often in the context of severe pneumonia requiring intensive care admission.

### Potential benefits of the Spotfire platform versus conventional quadriplex

Using a conventional quadriplex panel instead of the Spotfire platform would have missed 257 (43%) pathogens from 212 (39%) patients. Furthermore, of the 350 patients with positive Spotfire test and requiring hospital admission, all required isolation precautions. Indeed, even if the decision to place a patient in an isolation room may vary between institutions (as some centers choose not to isolate or to cohort patients infected with viruses considered less clinically significant, such as rhinovirus), in our institution, all patients with a positive Spotfire result were isolated according to infection-control policies: single rooms were prioritized whenever possible, and cohorting was only implemented. When single-room capacity was exceeded, strictly among patients infected with the same viral species (e.g., the same influenza subtype or SARS-CoV-2 variant). These patients were placed in single rooms (*n* = 301) or in double rooms (cohorting, *n* = 49). Among them, 158 (45%) were infected with at least one pathogen not included in the quadriplex panels.

Among the 540 patients with positive test results using the Spotfire platform, 328 (60.7%) were involved in the QV group, 44 (8.1%) in the AB group, and 168 (31.1%) in the NQV group. Oxygen therapy was required in 175 (53%) patients of the QV group, 27 (61.4%) in the AB group, and 100 (59.5%) in the NQV group (*P* = 0.318). Hospitalization was required in 212 (64.6%) patients of the QV group, 25 (56.8%) in the AB group, and 113 (67.3%) in the NQV group (*P* = 0.432). Thirty-day mortality rates were 7.3%, 6.8%, and 8.9%, respectively (*P* = 0.793) (Table 3). By contrast, significant differences were observed for the time from ED admission to medical decision (308 ± 247 min vs 395 ± 237 and 370 ± 222 min for AB, QV, and NQV, respectively; *P* = 0.009) and hospital length of stay (5.6 ± 4.0 days vs 10.7 ± 9.1 and 11.0 ± 9.7 days for AB, QV, and NQV, respectively; *P* = 0.007) (Table 3). Pairwise comparisons confirmed that AB patients had significantly shorter ED and hospital stays than both QV and NQV patients after Bonferroni correction, while no significant difference was observed between QV and NQV groups.

**TABLE 3:** Clinical outcomes according to pathogen group detected by Spotfire (*n* = 540)^*a*^

Characteristics	Quadriplex viruses (QV) (*n* = 328)	Non-quadriplex viruses (*n* = 168)	Atypical bacteria (*n* = 44)	*P*-value^*b*^
Oxygen therapy (via nasal cannula or face mask), *n* (%)	175 (53)	100 (60)	27 (61)	0.318
Medical decision-making time, min, mean ± SD	395 ± 237	370 ± 222	308 ± 247	**0.009** ^*c*^
Hospital admission, *n* (%)	212 (65)	113 (67)	25 (57)	0.432
Length of hospital stay, days, mean ± SD	10.7 ± 9.1	11.0 ± 9.7	5.6 ± 4.0	**0.007** ^*c*^
30-day mortality, *n* (%)	24 (7.3)	15 (8.9)	3 (6.8)	0.793

^*a*^ Data are presented as *n* (%) or mean ± SD. Pathogen groups were defined as follows: quadriplex viruses (QV) = detection of at least one virus included in standard quadriplex panels (SARS-CoV-2, influenza A/B, respiratory syncytial virus), regardless of co-infections; non-quadriplex viruses (NQV) = detection of respiratory viruses not included in quadriplex panels, with no co-detection of QV viruses or atypical bacteria; atypical bacteria = detection of at least one atypical bacterium (*Mycoplasma pneumoniae, Chlamydia pneumoniae, or Bordetella pertussis*), with or without co-infection with NQV viruses.

^*b*^ *P*-values correspond to overall group comparisons (Kruskal–Wallis for continuous variables and Chi-square for categorical variables, as appropriate). Bold *P*-values indicate statistically significant differences (*P* < 0.05).

^*c*^ In pairwise post-hoc comparisons, no significant difference was observed between quadriplex and non-quadriplex viral groups, whereas the atypical bacterial group was significantly different from both viral groups (*P* < 0.05).

## DISCUSSION

During the 14 weeks of the 2023 winter LRTIs outbreak season in France, 1,320 Spotfire tests were performed in adults presenting with LRTI symptoms at the ED of Poitiers University Hospital, with a reliable result return rate of 99.2% and a positivity rate of 41.2%, reflecting appropriate patient selection for testing. No nasopharyngeal swab was sent directly to the main laboratory during the platform’s opening hours, except for the 10 cases of failed results not resolved after repeat analysis. These findings confirm the feasibility of integrating this multiplex PCR platform into the routine workflow of a high-volume ED. Successful implementation was likely supported by a dedicated team of nurses, specially trained to operate the platform, without increasing the workload of the healthcare staff, highlighting the feasibility of the test when operated by non-laboratory clinicians. This organizational model likely facilitated the successful integration and may be critical for replication in other facilities. To our knowledge, this is the first real-world study to evaluate Spotfire implementation as a POC diagnostic tool in an ED. These results are consistent with previous evidence supporting the operational value of syndromic POC testing in acute care settings, particularly during periods of high viral circulation (21). Spotfire additionally demonstrated an analytical turnaround time under 20 min, and in our real-life ED setting, the overall delay from prescription to clinician result availability was 37 min (IQR 29.3–53.0, *n* = 810). This timeframe, which accounts for patient identification, sampling, and reporting, remained fully compatible with emergency workflows and represents a substantial improvement, considering that a recent systematic review pooling 15 studies reported mean turnaround times of 1536 min for standard virological testing and still 218 min for rapid multiplex PCR assays (22). Spotfire also allows the search of a much more complete viral panel and detection of atypical bacterial pathogens, both previously limited to main laboratories.

Our epidemiological results support the added diagnostic value of broad multiplex panels beyond the four usual viruses (the quadriplex panel SARS-CoV-2, influenza A/B, and RSV) targeted by existing POC tests. Notably, the 2023–2024 winter season coincided with an unusual worldwide resurgence of *Mycoplasma pneumoniae* infections, after several years of minimal circulation during the COVID-19 pandemic (19, 23). This epidemiological context likely contributed to the relatively high proportion of atypical bacterial detections in our cohort. From a broader perspective, this highlights one of the main advantages of multiplex syndromic testing: by maintaining a comprehensive panel, it allows early identification of unexpected or out-of-season pathogens, providing an effective sentinel function for emerging respiratory outbreaks. Among the 598 pathogens detected with the Spotfire, 257 (43.0%) would not have been identified by a standard quadriplex panel, such as recommended in national guidelines (18, 20). At the patient level, this corresponds to 212 (39.3%) positive patients who would have been tested negative. Among them, 44 (20.8%) were diagnosed with atypical bacterial infections, which may have required prompt and specific antibiotic treatment that could have been delayed or omitted in the absence of rapid syndromic testing. Conversely, in patients without atypical pathogens detected, prescriptions of macrolides or fluoroquinolones were infrequent (7.1%) and mostly limited to severe pneumonia cases requiring intensive care admission or to infections from other sources. These findings underline the dual clinical value of multiplex testing in both guiding targeted therapy and limiting unnecessary use of antibiotics against atypical bacterial pathogens.

Furthermore, all patients with documented respiratory viruses (including rhinovirus/enterovirus) should be isolated in single rooms or grouped together in double rooms for patients infected with the same pathogen (cohorting). Although often underestimated, rhinovirus has been associated with nosocomial outbreaks, supporting its inclusion in infection control protocols (24). The rapid turnaround time and broad detection spectrum of the Spotfire platform enabled early identification of infectious patients and facilitated the safe cohorting of 14% of hospitalized positive cases into double rooms with other patients carrying the same pathogen. Notably, among the 350 patients requiring isolation precautions, 42% would have been missed using quadriplex panels due to infections with viruses outside quadriplex targets. These findings underscore the operational value of expanded syndromic testing in optimizing patient placement and resource allocation in the ED, especially during high outbreak pressure when isolation capacities are constrained. Nevertheless, cohorting based on syndromic PCR results should be interpreted with caution. Although this approach optimizes bed allocation and limits nosocomial spread during peak epidemic activity, it may not fully prevent cross-infection between genetically related viruses (e.g., different seasonal coronaviruses or rhinoviruses/enteroviruses) and should be restricted to patients infected with the same viral species (e.g., same subtype of influenza virus or SARS-CoV-2 variant).

Patients with positive test results experienced shorter time to medical decision compared with those with negative tests. They were also less frequently hospitalized and had shorter hospital length of stay. While these differences may appear modest in absolute terms, they suggest clinically meaningful improvements in patient flow and resource allocation in a high daily patient-volume ED. One plausible explanation is that early microbiological documentation (particularly when supporting viral origin) enhanced clinical confidence, enabling faster and more appropriate decision-making. In line with this interpretation, patients with positive test results underwent significantly fewer additional investigations, including blood analyses and imaging, further supporting the role of rapid syndromic testing in reducing unnecessary diagnostic burden. This interpretation is consistent with prior findings from the ResPOC randomized trial, which demonstrated that POC syndromic PCR testing was associated with improved triage and shorter hospital stays (25). Similarly, other authors reported enhanced test turnaround and more effective isolation workflows with rapid diagnostics in the ED (26).

The rationale for recommending narrow pathogen panels, such as quadriplex assays, typically relies on a combination of factors, including high prevalence, clinical severity of these infections, and cost-effectiveness logic favoring limited but high-yield testing. Nevertheless, in our study, we found no difference in 30-day mortality, hospital admission rates, or need for oxygen therapy between patients infected with quadriplex viruses, atypical bacteria, or non-quadriplex viruses. Similarly, no difference was observed between quadriplex and non-quadriplex virus groups regarding medical decision-making time in the ED or hospital length of stay. These results challenge the assumption that non-quadriplex respiratory viruses are usually less aggressive and instead suggest that broader diagnostic coverage may be warranted, particularly in hospitalized or high-risk patients, which is consistent with recent evidence that some non-quadriplex viruses, such as rhinoviruses, can cause severe LRTIs even in immunocompetent adults (24). Importantly, 44 patients (8.1% of those with positive test) were diagnosed with atypical bacterial infections (representing 7.5% of all pathogens detected), which would have remained undocumented in this adult cohort using a viral-only panel. This reinforces the clinical value of expanded multiplex testing to guide not only isolation strategies but also appropriate antimicrobial therapy.

Taken together, these findings underscore the clinical relevance of syndromic testing in identifying a wide range of respiratory pathogens with potential implications for patient management. This is particularly aligned with the 2025 update of the French Infectious Diseases Society (SPILF) guidelines (18), which recommend the use of high multiplex RT-PCR panels for etiological diagnosis of community-acquired pneumonia (CAP), especially when atypical pathogens are suspected or when microbiological documentation is likely to influence therapeutic decisions. While routine use in all patients is not currently advocated, the guidelines emphasize their relevance during outbreak seasons and in patients requiring hospital admission. Our results support this approach by demonstrating that expanded syndromic testing can improve diagnostic yield and assist in real-time decision-making regarding both antibiotic use and infection control.

Nevertheless, this study has several limitations. First, the absence of a contemporaneous control group without access to molecular testing during the same period and operating hours limits the ability to draw causal inferences. This was not a randomized study but rather a real-life implementation of the Spotfire platform to support medical decision-making and optimize patient placement in the ED. Second, patient inclusion was based on clinical judgment and staff availability, reflecting real-life ED practice but potentially introducing selection bias. In this context, most included patients were elderly, consistent with our focus on individuals presenting with respiratory symptoms and requiring hospitalization. Older adults, who more often exhibit severe manifestations or underlying frailty, are therefore naturally overrepresented among hospitalized patients with LRTIs, which reinforces the external validity of our findings. Interestingly, patients with a positive Spotfire result more frequently required oxygen therapy but had shorter hospital stays and lower mortality rates. Several factors may contribute to this finding. A selection effect cannot be excluded, as patients with confirmed viral infections were generally younger and less comorbid than those with negative results. Moreover, the rapid identification of a viral pathogen could have reduced diagnostic uncertainty, limiting unnecessary additional investigations and enabling earlier de-escalation of empirical treatments. This more focused clinical management may have contributed to shorter hospital stays and improved overall care efficiency. Third, data on comorbidities were extracted from unstructured medical records, and no standardized severity scores were recorded, preventing adjustment for case mix and increasing the risk of residual confounding. Fourth, the Spotfire platform was only available from 10 a.m. to 10 p.m., potentially excluding some eligible patients during night hours. Extending availability to a 24/7 model may further enhance its clinical and operational utility. However, the number of patients presenting overnight was limited, and in practice, many of them waited until morning to benefit from rapid Spotfire testing once the platform became available. This resulted in a noticeable peak of analyses during the first hours of operation each day, effectively minimizing the potential temporal selection bias. The Spotfire system was implemented dynamically during the 2023–2024 epidemic period, with activation triggered by the laboratory’s detection of the onset of increased respiratory pathogen circulation through local epidemiological surveillance. This adaptive approach ensured optimal resource use while maintaining rapid diagnostic availability during periods of high incidence. The optimal deployment strategy, whether continuous year-round or guided by epidemiological triggers defined in close collaboration between microbiologists and clinicians, remains to be determined. Larger-scale studies are warranted to assess which approach provides the best balance between diagnostic benefit, operational feasibility, and antimicrobial stewardship. Fifth, all microbiological analyses were performed on nasopharyngeal swabs rather than lower respiratory tract specimens. Although lower respiratory tract samples may theoretically better reflect LRTI etiology, nasopharyngeal swabs have shown substantial agreement with bronchoalveolar lavage for pathogen detection in adult pneumonia (7). However, this methodological choice inherently limits bacterial documentation, as common etiological agents of LRTIs, such as *Streptococcus pneumoniae* or *Haemophilus influenzae*, are not reliably identified by nasopharyngeal PCR alone. Consequently, our study primarily describes viral and atypical bacterial epidemiology but provides limited insights into the prevalence of typical bacterial pathogens and their impact on antibiotic stewardship. Finally, no formal cost-effectiveness analysis was conducted, and we did not assess the potential impact on healthcare-associated infection rates. Such evaluation would be challenging, as those infections have multifactorial origins. This remains an important avenue for future research to inform sustainable implementation strategies in emergency care.

Despite these limitations, our findings add to the growing body of evidence supporting the role of real-time syndromic testing in improving patient management and resource optimization in acute care settings. Previous studies evaluating rapid or near-POC multiplex molecular testing performed in laboratory settings with short turnaround times of a few hours have already demonstrated clinical and operational benefits, including faster diagnostic confirmation, improved patient isolation, and optimized antimicrobial therapy (14, 22, 27–29). Our findings are consistent with these results, confirming that rapid molecular syndromic testing enhances patient management in acute care. Importantly, our study further extends this evidence by demonstrating that comparable benefits can be achieved with a true multiplex POC platform implemented directly within the ED, achieving a total turnaround time of less than 1 h. Prospective, multicenter studies including randomized controlled trials, pediatric cohorts, and cost-effectiveness analyses are needed to formally evaluate the clinical and economic impact of multiplex POC testing in emergency care.

### Conclusion

To conclude, the Spotfire multiplex PCR testing was successfully implemented in a high-volume ED during a period of outbreak pressure. Its deployment was supported by a trained nursing team operating the platform directly in the ED. While limited to 10 a.m.–10 p.m., this organizational model proved compatible with routine workflows and enabled standardized, high-throughput testing. Implementation of this platform in EDs should improve patient flow, antimicrobial decisions, and isolation strategies beyond quadriplex testing.

## Data Availability

De-identified participant data supporting the findings of this study are available from the corresponding author upon reasonable request. In accordance with French data protection regulations (CNIL MR-004), data cannot be made publicly available. Tables S1 to S5 are provided in the supplemental material.

## SUPPLEMENTAL MATERIAL

Supplemental material jcm.01313-25-s0001.docx Detailed distributions of detected pathogens (single and co-infections) and the comparison of laboratory and imaging prescriptions according to Spotfire test results.

ASM does not own the copyrights to Supplemental Material that may be linked to, or accessed through, an article. The authors have granted ASM a non-exclusive, world-wide license to publish the Supplemental Material files. Please contact the corresponding author directly for reuse.
