# Supplementary material for: Impact of multiplex PCR point-of-care platform implementation for respiratory pathogen detection in an emergency department with high daily patient volume
Source: J Clin Microbiol. 2025 Dec 1;64(1):e01313-25. doi: 10.1128/jcm.01313-25 (PMC12802140; doi:10.1128/jcm.01313-25)
Supplement: Supplemental material — Detailed distributions of detected pathogens (single and co-infections) and the comparison of laboratory and imaging prescriptions according to Spotfire test results. [file jcm.01313-25-s0001.docx]

# Supplementary materials

**Impact of Multiplex PCR Point-of-Care Platform Implementation for Respiratory Pathogens Detection in a High Daily Patient-Volume Emergency Department**

**Authors**

**Benjamin BIGAUD^1,2^, Nicolas MARJANOVIC^1^ ; Luc DEROCHE^4,5,6^ ; Bertrand DRUGEON^1,2,3^ ; Marvin PIOT^1^ ; Nicolas LEVEQUE^4,5^ ; Olivier MIMOZ^1,2,3^ ; Jérémy GUENEZAN^1,2,3^**

**1** Service des Urgences SAMU SMUR, CHU Poitiers, France;

**2** INSERM U1070, Pharmacologie des Agents Anti-Infectieux et Résistance (PHAR2), Poitiers, France;

**3** Alliance for Vascular Access Teaching and Research (AVATAR) group, Griffith University, Nathan, Queensland, Australia;

**4** Laboratoire de virologie et mycobactériologie, CHU de Poitiers, France;

**5** Laboratoire Inflammation Tissus Epithéliaux et Cytokines, Université de Poitiers, 86073 Poitiers, France;

**6** INSERM U1313, Ischémie Reperfusion, Métabolisme et Inflammation Stérile en Transplantation (IRMETIST), Poitiers, France.

## Table S1. Distribution of pathogens detected in all the 540 positive patients

|  | Number of pathogens detected  (n = 598 in total) |
| --- | --- |
| *Influenza A* | 185 (30.9%) |
| *Rhinovirus/enterovirus* | 126 (21.1%) |
| *SARS-CoV-2* | 112 (18.7%) |
| *Seasonal coronavirus* | 46 (7.7%) |
| *Respiratory syncytial virus* | 43 (7.2%) |
| *Mycoplasma pneumoniae* | 43 (7.2%) |
| *Human metapneumovirus* | 27 (4.5%) |
| *Parainfluenzae* | 10 (1.7%) |
| *Adenovirus* | 3 (0.5%) |
| *Bordetella pertussis* | 1 (0.2%) |
| *Influenza B* | 1 (0.2%) |
| *Chlamydia pneumoniae* | 1 (0.2%) |

Results are n (%). SARS-CoV-2 denotes *severe acute respiratory syndrome-related coronavirus 2*

## Table S2. Distribution of pathogens in the 485 included patients with a single pathogen detected

|  | Number of pathogens detected  (n = 485 in total) |
| --- | --- |
| *Influenza A* | 160 (33.0%) |
| *Rhinovirus/enterovirus* | 93 (19.2%) |
| *SARS-CoV-2* | 92 (19.0%) |
| *Mycoplasma pneumoniae* | 37 (7.6%) |
| *Seasonal coronavirus* | 36 (7.4%) |
| *Respiratory syncytial virus* | 34 (7.0%) |
| *Metapneumovirus* | 22 (4.5%) |
| *Parainfluenzae* | 7 (1.4%) |
| *Adenovirus* | 3 (0.6%) |
| *Chlamydia pneumoniae* | 1 (0.2%) |

Results are n (%). SARS-CoV-2 denotes *severe acute respiratory syndrome-related coronavirus 2*

## Table S3. Distribution of pathogens in the 52 included patients with two pathogens detected

| Pathogen combination | Number of combinations  (n = 52 in total) |
| --- | --- |
| *Influenza A + rhinovirus/enterovirus* | 11 (21.2%) |
| *Rhinovirus/enterovirus + SARS-CoV-2* | 6 (11.5%) |
| *Influenza A + SARS-CoV-2* | 5 (9.6%) |
| *Rhinovirus/enterovirus + Mycoplasma pneumoniae* | 4 (7.7%) |
| *Rhinovirus/enterovirus + metapneumovirus* | 3 (5.8%) |
| *Parainfluenza + rhinovirus/enterovirus* | 3 (5.8%) |
| *Respiratory syncytial virus + SARS-CoV-2* | 3 (5.8%) |
| *Respiratory syncytial virus + rhinovirus/enterovirus* | 3 (5.8%) |
| *Seasonal coronavirus + SARS-CoV-2* | 3 (5.8%) |
| *Influenza A + seasonal coronavirus* | 3 (5.8%) |
| *Influenza A + respiratory syncytial virus* | 2 (3.8%) |
| *Rhinovirus/enterovirus + seasonal coronavirus* | 1 (1.9%) |
| *Influenza A + metapneumovirus* | 1 (1.9%) |
| *Influenza A + influenza B* | 1 (1.9%) |
| *Seasonal coronavirus + Bordetella pertussis* | 1 (1.9%) |
| *Metapneumovirus + Mycoplasma pneumoniae* | 1 (1.9%) |
| *Mycoplasma pneumoniae + SARS-CoV-2* | 1 (1.9%) |

Results are n (%). Percentages indicate the number of patients with each pathogen combination among all patients with two pathogens detected (n = 52). SARS-CoV-2 denotes *severe acute respiratory syndrome-related coronavirus 2*

## Table S4. Distribution of pathogens in the 3 included patients with 3 pathogens detected

| Pathogen combination | Number of combinations  (n = 3 in total) |
| --- | --- |
| *Influenza A + enterovirus/rhinovirus + coronavirus* | 1 (33.3%) |
| *Influenza A + coronavirus + SARS-CoV-2* | 1 (33.3%) |
| *RSV + rhinovirus/enterovirus + SARS-CoV-2* | 1 (33.3%) |

Results are n (%). RSV denotes Respiratory syncytial virus, and SARS-CoV-2 *severe acute respiratory syndrome-related coronavirus 2*

Table S5. Prescription of laboratory and imaging investigations according to Spotfire® test results

|  | All patients (n=1310) | Positive test  (n=540) | Negative test  (n=770) | Absolute difference | *p-value* |
| --- | --- | --- | --- | --- | --- |

| Laboratory tests  Complete blood count  Serum electrolytes/creatinine  D-dimer  Lactate  Arterial blood gas  Pneumococcal urinary antigen  Legionella urinary antigen  Imaging test  Chest X-ray  Chest CT-scan | 1,188 (91)  1,187 (91)  192 (15)  688 (53)  641 (49)  135 (10)  131 (10)  935 (71)  136 (10) | 467 (86)  466 (86)  101 (19)  292 (54)  278 (51)  64 (12)  61 (11)  404 (75)  58 (11) | 721 (94)  721 (94)  91 (12)  396 (51)  363 (47)  71 (9.2)  70 (9.1)  531 (69)  78 (10) | -7.2 [-10.5 to -3.8]  -7.3 [-10.7 to -4.0]  +6.9 [2.9 to 10.9]  +2.6 [-2.8 to +8.1]  +4.3 [-1.2 to +9.8]  +2.6 [-0.8 to +6.0]  +2.2 [-1.1 to +5.6]  +5.9 [0.9 to 10.8]  +0.6 [-2.8 to 4.0] | <0.001  <0.001  <0.001  0.3  0.12  0.12  0.2  0.021  0.7 |
| --- | --- | --- | --- | --- | --- |

Values are presented as n (%). Differences between groups are reported as absolute differences with 95% confidence intervals.
Bold *p*-values indicate statistically significant differences (*p* < 0.05).
